# Ten simple rules for preparing and delivering effective funding pitches in competitive grant applications

**DOI:** 10.1371/journal.pcbi.1014840

**Published:** 2026-10-07

**Authors:** Andy Göbel, Tilman D. Rachner

**Affiliations:** 1 Division of Endocrinology and Metabolic Bone Diseases, Department of Medicine III, University Hospital Carl Gustav Carus, Technische Universität Dresden, Dresden, Germany; 2 German Cancer Consortium (DKTK), partner site Dresden, and German Cancer Research Center (DKFZ), Heidelberg, Germany; Dassault Systemes BIOVIA Ltd, UNITED STATES OF AMERICA

## Abstract

In grant applications, oral presentations can be an integral part of the evaluation process. Funding pitches are a specific form of pitch presentations delivered during competitive review procedures to complement a written proposal. Their goal is to convince reviewers of both your project's strength and excellence as well as your suitability as a researcher. This paper offers ten simple rules on how to prepare and deliver effective funding pitches, with a particular focus on content selection, clear structuring, and convincing scientific argumentation. We also highlight the importance of practice and feedback and share practical strategies on how to handle questions and critical discussion professionally.

## Introduction

Grant applications are an essential part of a scientific career. Although written proposals remain the central component of most application processes, some funding organizations and institutional programs additionally invite selected applicants to present their project orally in front of a review panel, typically followed by questions and critical discussion. These funding pitches provide applicants with an opportunity to clarify the central idea of their proposal, emphasize its relevance and feasibility, and demonstrate their ability to conduct the planned research. Their primary purpose is to support the assessment of the proposed project and the applicant within a formal funding decision.

Often, pitch talks, also known as “elevator pitches,” are the format of choice, as they require you to convince your audience within the time span of an average elevator ride (usually 1–5 min). Funding pitches may differ in duration, presentation setting, available media, evaluation criteria, career stage, and project scale, depending on the specific funding process. A pitch for an intramural early-career grant may therefore require a different emphasis than a presentation for a larger collaborative proposal or a topic-specific funding call. When preparing the pitch, the specific requirements and evaluation criteria of the respective funding program should therefore always be considered.

The ten rules presented here combine principles from the literature on scientific presentations, science communication, and research pitching with our practical and teaching experience with funding-related presentations. Rather than being derived from a formal systematic review, the rules synthesize recurring principles that we considered most relevant to the preparation and delivery of short funding pitches. They were organized to broadly follow the preparation process, from understanding the purpose and review panel to developing the content, presentation, and delivery. They are intended as a checklist for researchers who have submitted a grant proposal and are now preparing to present and discuss their project with a review panel. The rules should help applicants identify the content, arguments, and presentation strategy most relevant to their particular funding context.

## Before you start – Understand the purpose of a funding pitch

In general, presentations serve four common purposes: *to inform or educate*, *to convince or persuade*, *to inspire or motivate*, and *to entertain* [1]. Pitch talks combine these elements with a particular focus on presenting a project idea in a concise and convincing way. Indeed, pitch talks are a powerful communication tool increasingly used in the context of grant applications, interdisciplinary collaborations, and innovation-driven environments [2,3].

Their specific purpose, however, depends strongly on the context in which they are delivered. In a funding context, a pitch should convince a review panel that the proposed project is relevant, scientifically sound, feasible, and worth supporting, and that the applicant is well suited to conduct it.

Funding pitches are typically very *short* (1–5 min), *sharply focused*, and *precise* (Fig 1). Importantly, they are not simply condensed versions of standard presentations. Rather than providing a comprehensive overview of the written proposal, they require applicants to identify its most important elements, present strong and well-supported arguments, and make the relevance of the proposed research immediately clear. An effective funding pitch should also provide a strong basis for the subsequent discussion, in which reviewers can explore scientific details, limitations, risks, feasibility, and other aspects of the proposal.

**Fig 1:**
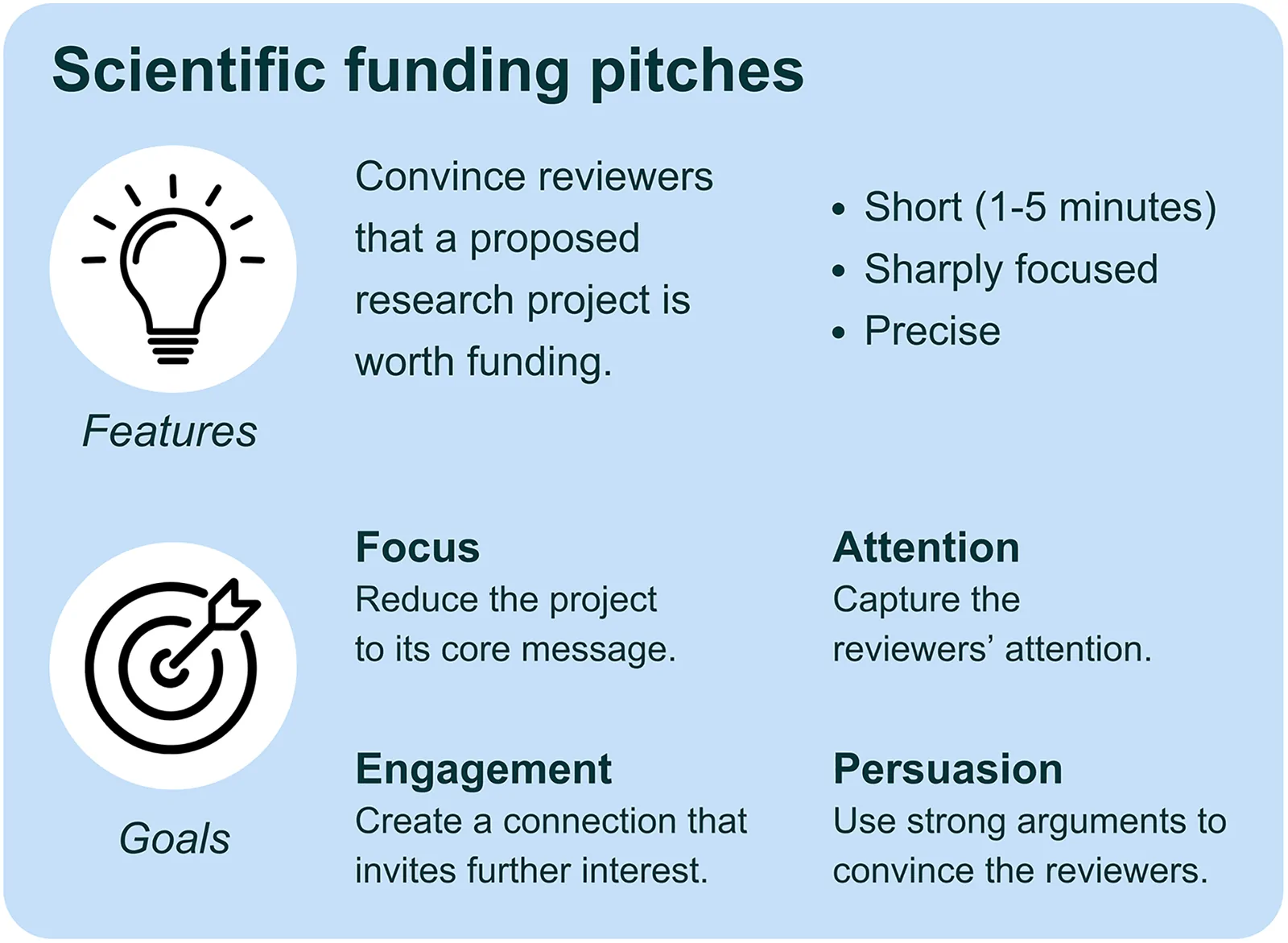
Features and goals of scientific funding pitches.

One frequently cited historical example illustrates the basic idea behind pitching. In the middle of the 19th century, elevator use was considered highly risky due to frequent malfunctions and fatal falls [4]. At the 1854 World’s Fair in New York, the American inventor Elisha Otis constructed a visible elevator shaft in front of a live audience. While standing on top of the platform, he instructed his assistant to cut the supporting rope. As the platform began to fall, panic spread through the crowd until the safety brakes triggered and brought the platform to a sudden halt before reaching the ground. Otis, enjoying the dramatic effect of his demonstration, announced: “All safe, gentlemen. All safe.” The public perception of elevators was revolutionized and just recently, the Otis Elevator Company celebrated its 170th anniversary, an achievement that, at least in part, originates from Elisha Otis’s memorable demonstration [4–6].

This example illustrates that an effective pitch starts from a relevant problem, makes clear why it matters, and demonstrates how the proposed solution addresses it. In a funding pitch, this should be communicated by explaining why a scientific problem deserves attention, how the proposed project will address it, and why the applicant and approach are ideally suited.

## Rule 1 – Analyze the review panel

Funding pitches are prepared for the review panel, not for the applicant. Before you start working on the content, consider who will evaluate your project and what they need to assess it [7,8]. The content and tone of your funding pitch should be carefully tailored to the specific audience. Four specific questions help you define it [9]:

### First: Who are they?

A review panel often consists of basic and clinical scientists, mostly professors or at least researchers with solid and long-standing experience. Depending on whether you are pitching for an external third-party funder or for internal institutional calls, reviewers may be local, national, or international invited experts. In addition, deciding representatives of the funding organization itself may participate in the selection process.

### Second: What is their level of expertise in your scientific field?

A review panel must be composed of qualified people to assess the quality of your project as well as your eligibility as a researcher. However, even highly qualified reviewers with strong expertise in related approaches may not be equally familiar with all the details of your work, specific methods, technologies, or concepts, particularly when the panel includes experts from adjacent scientific fields [10]. Regardless of their background, always adapt your explanations and terminology according to the reviewers’ expertise and consider their perspective [11]. Always be prepared for reviewers who are unfamiliar with your topic [3]. For example, clinicians may benefit from a brief reminder of specific mechanistic concepts, whereas basic researchers may require additional context on clinical challenges in daily patient care. The goal is not to oversimplify the science, but to provide all reviewers with the information they need to follow and evaluate your arguments.

### Third: How do the listeners feel about you and your topic?

In the ideal scenario, reviewers are already interested in your topic, familiar with your field, and convinced by important aspects of your written proposal. In that case, you may be able to focus more strongly on specific details, your unique approach, or preliminary data. However, reviewers may also be indifferent, skeptical, or even critical about individual aspects of your hypothesis, approach, feasibility, or your suitability as the ideal applicant. Particularly in such cases, it is important to establish your credibility early, clearly explain the relevance of your project, and provide well-reasoned arguments that directly address potential concerns.

### Fourth: What is the goal of the listeners?

In funding, reviewers aim to identify the most promising projects that meet the objectives and evaluation criteria of the respective funding program and to assess whether the applicants are qualified to conduct them successfully. They also need to evaluate whether the proposed project is scientifically sound, feasible, and likely to make effective use of the requested funding.

### But how can you analyze the listeners?

If the names of the reviewer panel are publicly available, professional information can help you understand their scientific background, expertise, and research interests. Useful sources include institutional and laboratory websites, scientific publications and PubMed records, as well as publicly available professional profiles, interviews, press articles, or presentations [4]. If colleagues have previously interacted with members of the panel in a professional context, they may also be able to provide information about their scientific interests or the types of questions they commonly raise [12].

If the reviewers are unknown in case of blinded or confidential review processes, prepare for a heterogeneous panel rather than trying to predict individual personalities, characteristics, or preferences. Consider different scientific backgrounds and levels of familiarity with your topic and ask colleagues from both within and outside your research field to review your pitch and challenge you with questions. Such rehearsals can reveal assumptions, unclear explanations, or potential questions that you may otherwise overlook (see **Rule 8**).

Analyzing the audience is a key consideration in scientific communication [10,13,14]. Being conscious of the audience’s expertise, background, attitudes, and goals will help you make better decisions in terms of background information, terminology, and most effective arguments. It will also shape how you communicate your message clearly and convincingly [14].

## Rule 2 – Define your key messages

The message of a funding pitch is never “*Please, support my project with your funding*”. This is the goal of your pitch, as it defines what you would like to achieve [15]. In addition to this main goal, you might pursue some personal objectives such as sparking general curiosity about your topic, receiving constructive feedback or new ideas, or leaving a memorable impression as a speaker and researcher*.*

In a funding pitch, three key messages should be clearly and effectively delivered:

- The problem is relevant and needs to be solved.
- The proposed solution is feasible and will make an impact.
- I am well qualified to carry out the project [16].

While these three key messages provide a general framework for funding pitches, they should be adapted to the specific project, funding call, and evaluation criteria and supported by appropriate evidence. Beyond the three key messages, it is helpful to define one clear take-home message that you want the review panel to remember, a concise statement summarizing the central idea of your project and the expected impact.

## Rule 3 – Describe the scientific problem & present the solution

It is essential to reduce the complexity of your projects to its core. One of the first critical considerations is: *Why should the review panel care at all?* To engage the reviewers, they must understand why your project is relevant. In essence, they need to understand what problem your research is trying to solve and why it needs to be solved now [16] (Table 1). Clarifying which category your project addresses helps to evaluate its relevance [2]. Avoid vague or overly broad problem statements and define the problem as precisely as possible. Be specific, identify its underlying cause, clarify who or what is affected, consider potential barriers, and above all, explain why the problem matters now (Fig 2). A clearly stated problem increases credibility and helps the review panel understand the relevance and urgency of your project from the very beginning.

**Table 1:** Scientific problems.

Category	Definition	Example
Technical/methodological problems	Limitations in tools, reproducibility, practical implementation	Lack of noninvasive, real-time imaging techniques for detecting micrometastases in certain malignancies
Mechanistic/pathway-related problems	Gaps in understanding biological mechanisms or causal pathways	Unknown role of glial cell-derived factor XY in neurodegeneration in patients with Alzheimer´s disease
Therapeutic/clinical problems	Challenges in developing, optimizing, or applying effective treatments	Tumor movement in pancreatic cancer reduces the accuracy and effectiveness of radiation therapy
Societal/environmental problems	Issues with social, ethical, or environmental relevance	Anthropogenic destruction of habitats leads to extinction of multiple species
Predictive problems	Challenges in predicting biological processes, disease progression, or treatment response	Lack of biomarkers to predict the response of checkpoint inhibitors in cancer therapy
Data/evidence problems	Lack of sufficient, reliable, or representative data	Incomplete surveillance data on antibiotic resistance in vulnerable populations
Translational problems	Barriers in moving from basic research to applied solutions	Limited transferability of promising preclinical cancer therapies into effective treatments for patients.

**Fig 2:**
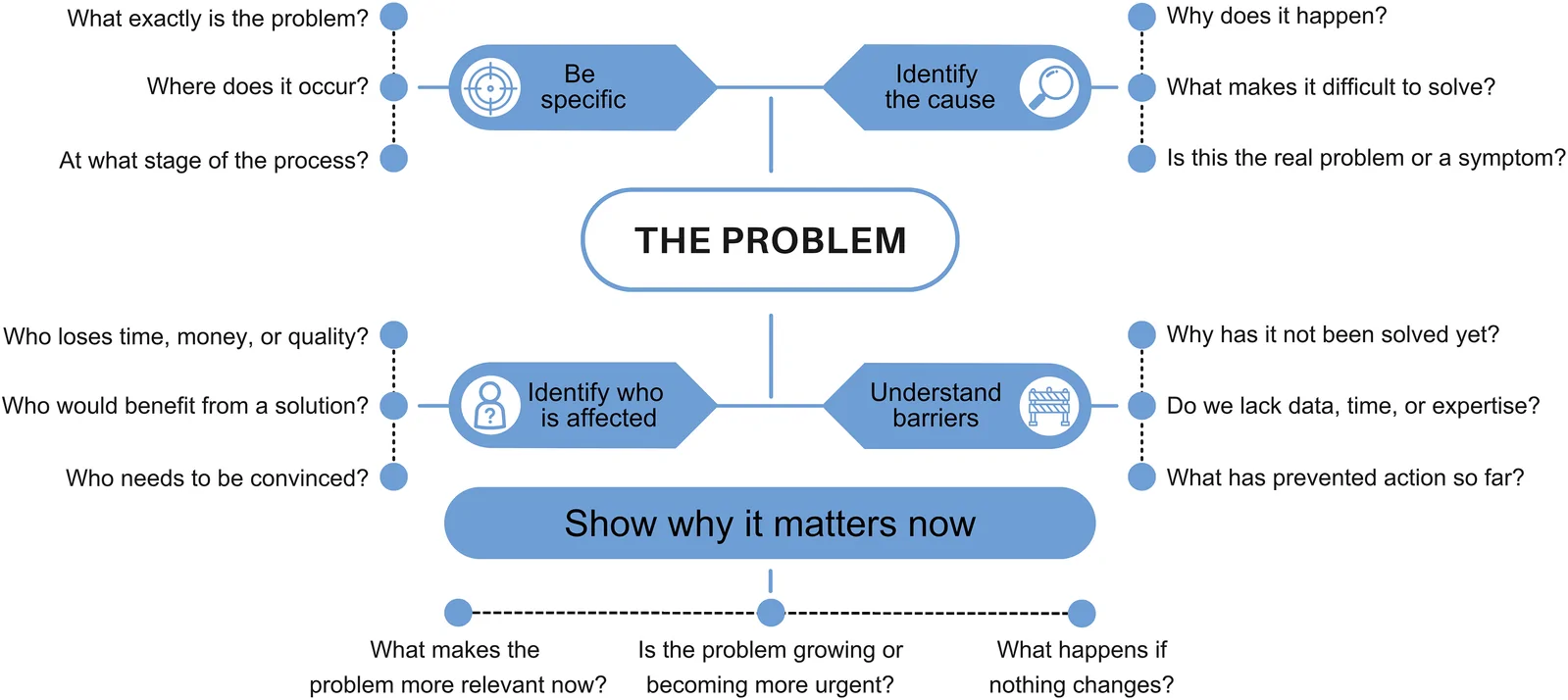
Defining the scientific problem. The framework highlights key questions to specify the problem, identify its causes and affected stakeholders, understand existing barriers, and clarify why the problem requires attention now.

The next key question is: *How is your research contributing to solving the problem?* At this stage, put your approach in a broad context and write out the full strategy, including every single effort that contributes to the solution, beyond wet lab experiments. Your approach may include *in vitro*, *in vivo, and in silico* experiments, clinical data, patient cohorts, novel technologies, or other methodological and conceptual approaches that are essential to your project.

## Rule 4 – Collect convincing arguments

Having defined the problem and your proposed solution, you should now collect strong *arguments* that emphasize why your project deserves funding, why your approach is suitable to address the problem, and why you are the ideal candidate to successfully conduct the project (Table 2). Select and prioritize these arguments according to the objectives and evaluation criteria of the respective funding call. Persuasive arguments should be supported by evidence and should openly acknowledge relevant uncertainties, limitations, and risks of the proposed approach.

**Table 2:** Categories of arguments in funding pitches.

Project-related	Solution-related	Qualification-related
Matches FINER criteria	Novel methodology	Methodological expertise
Alignment with the objectives of the funding call	Complementary partner expertise	Scientific background and experience
Expected impact / added value	Interdisciplinary approach	Established networks
Potential for publication and follow-up projects	Clinical and / or industrial application	Career development perspective
Potential for collaborations	Sustainability	Motivation and commitment

*Project-related arguments* include the feasibility and expected impact of your project as well as its potential to generate new knowledge, publications, follow-up projects, or collaborations. Define how your project fills a gap in knowledge, establishes novel concepts, and advances the field. To reflect on these dimensions, you may use the FINER criteria (Fig 3). These criteria define whether your project is feasible, interesting, novel, ethical, and relevant, help evaluate your research question and collect convincing arguments [17]. Moreover, they may reveal unseen or neglected requirements and help define the broader relevance of the planned study, for example, its potential impact on society, science, and economy [11].

**Fig 3:**
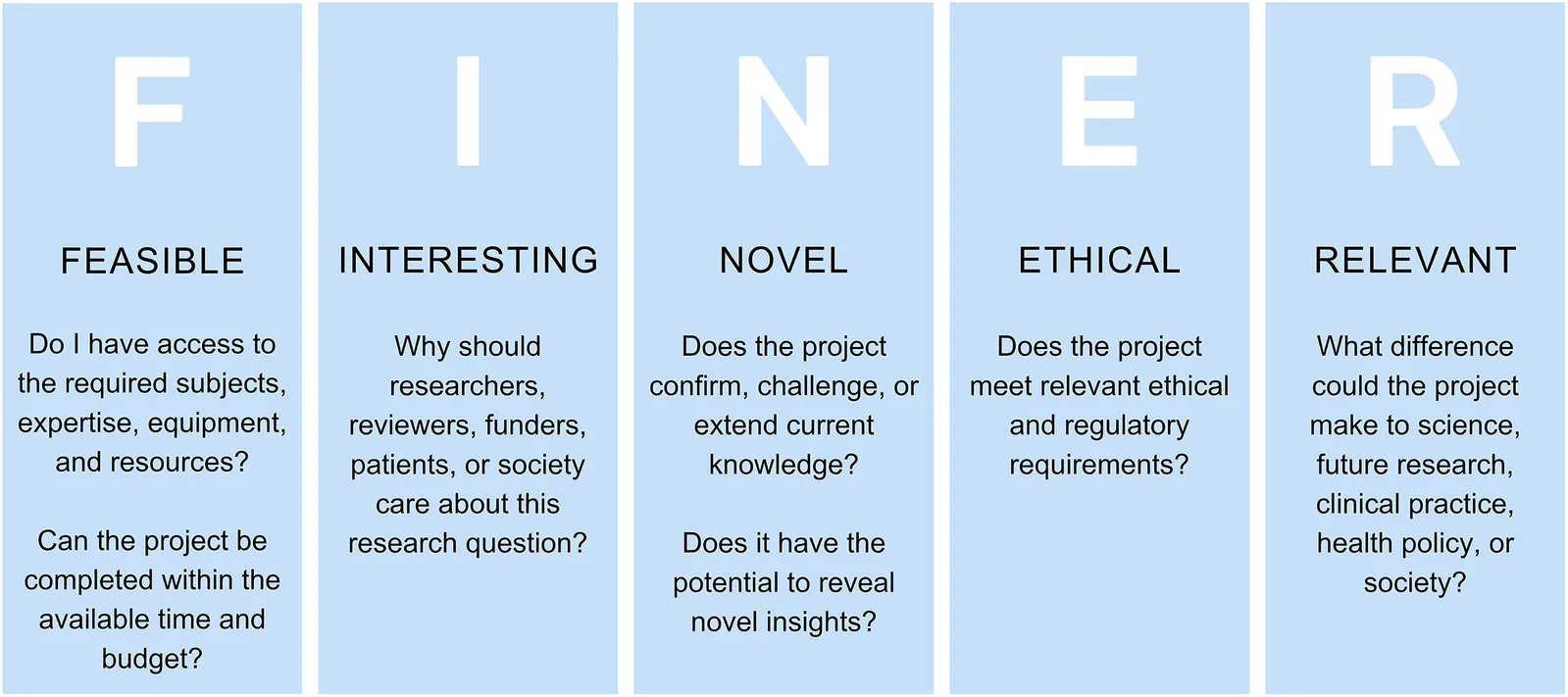
The FINER criteria. Questions to identify key arguments for your funding pitch. The FINER criteria define whether your project is feasible, interesting, novel, ethical, and relevant, help evaluate your research question and collect supportive arguments [17].

*Solution-related arguments* reinforce the strengths and originality of your solution and should focus on aspects that go beyond standard approaches. These may include key preliminary data, emerging technologies, establishment of novel methods, synergies between different disciplines and partner expertise, translational aspects, novel concepts, industrial or clinical application, or sustainability. Select two or three particularly convincing components of your approach and keep further details for discussion.

*Qualification-related arguments* establish and strengthen your credibility as a suitable researcher to conduct the project [15]. These include your academic background, experience with the topic, methodological expertise, preliminary work, and established networks within the scientific community. In addition, clearly communicating your motivation and future career perspective can be excellent indicators of your commitment [16]. Consider how the proposed project builds on your previous work and contributes to your further scientific development.

Finally, tailor the selection and adjust the emphasis of your arguments according to the nature of the funding call. Programs for early-career scientists may focus more strongly on the applicant’s qualifications, whereas larger collaborative or translational proposals may require a sharper focus on feasibility, novelty, innovation, translation, application, and cooperation.

## Rule 5 – Make use of a compelling structure

Now develop a clear and thoughtful structure that will help you make the most of your limited time. When writing a scientific paper, we typically follow a standard sequence: *introduction → materials and methods → results → discussion → conclusion*. Many scientists unconsciously copy the same sequence for oral presentations. Particularly in funding pitches, using a “paper-like” structure will certainly fail to deliver your message effectively and to engage the review panel [4]. As the written proposal is usually already available to the reviewers, the funding pitch should not simply reproduce its content. Instead, use the limited presentation time to emphasize those aspects that are most relevant for the funding decision. The selection and structure of your content should therefore reflect the objectives and evaluation criteria of the respective funding call.

An effective approach is the “Speech Structure Building” [18] which is inspired by a building with different rooms and areas, each serving a clear function (Fig 4). Translate this to your funding pitch: You enter the basement with the introduction and problem, move into the core rooms presenting your proposed solution or approach and key arguments, and finish on the rooftop with a concise summary and key message before closing with the expected impact and a final statement.

**Fig 4:**
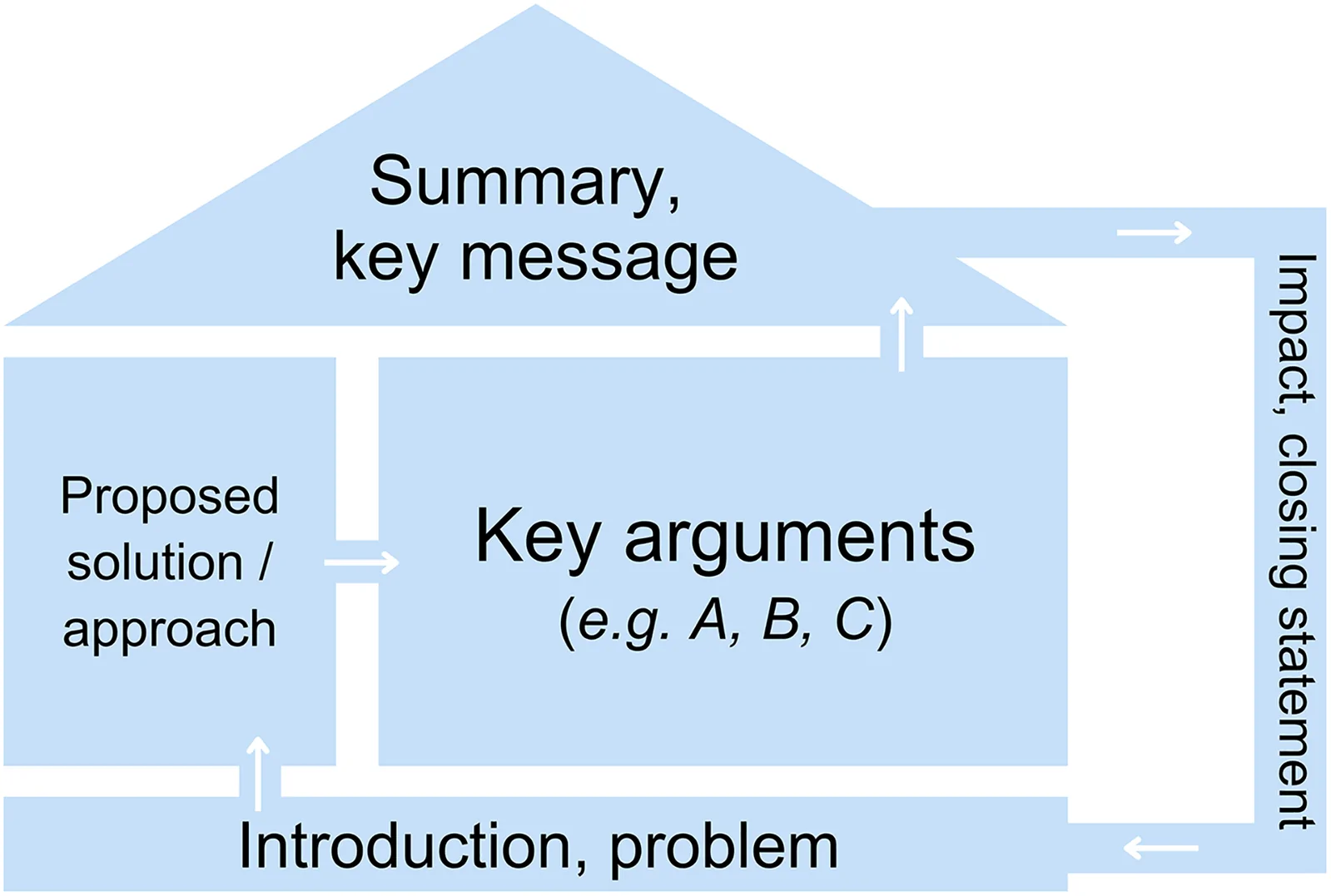
The Speech Structure Building. The structure is based on the Speech Structure Building concept described by Mück [18].

The basement of the “building” is your *introduction*. Start with a strong opening that immediately captures the audience’s attention, is engaging and relatable, and ideally connects to the problem your research aims to solve. This helps make the relevance of your project clear from the beginning [3,19] (Fig 5).

**Fig 5:**
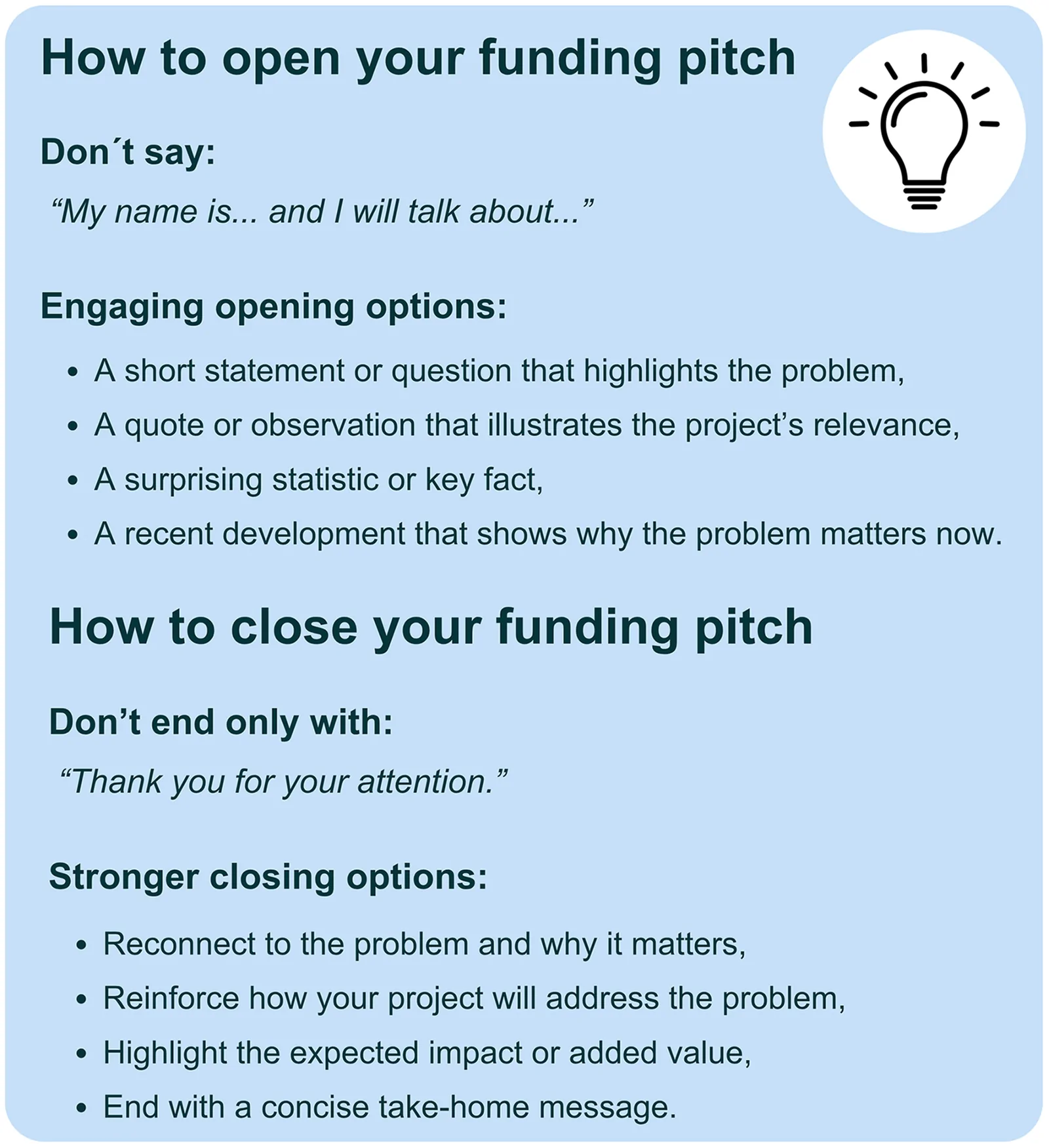
Options on how to open and close your funding pitch.

The next aspect that should be explained early is the *problem* your project will address and why it matters. Provide only tailored background information that makes the nature and urgency of the problem clear.

In the main rooms of your talk, you present your proposed *solution*, followed by the strongest *arguments* for why your project should be funded and why you and your team are well suited to conduct it. Prioritize those arguments that are most relevant to the funding call and that best demonstrate the strengths, feasibility, and expected impact of your project. Essential evidence and key arguments should be presented during the pitch rather than withhold for the discussion, as the corresponding questions may never be asked. Additional details, supporting analyses, and backup data can instead be prepared for the subsequent discussion.

The *finishing* of your talk must be strong and engaging. At this point, briefly summarize the problem and link it back to why your approach is suited to address it. Take the “drainpipe” back to your initial statement to memorably round off the talk. This closing loop illustrates how your project will help solve the problem, advance the field, or benefit stakeholders, and leaves the review panel with a clear impression of relevance and purpose. Clearly present the expected impact of your project and finish with a concise closing statement that leads naturally into the subsequent discussion (Fig 5).

Do not distribute your presentation time evenly across all sections. Keep general background information concise and reserve most of the available time for your proposed approach, the strongest supporting arguments, and those aspects that are particularly relevant to the funding decision.

Once you have defined your content and created a structure, carefully screen your pitch draft for dispensable information, including extended background details, complete methodological explanation, unrelated context, the entire list of collaborations, infrastructure or budget specifics, and extended acknowledgement [20]. Keep only the information required to understand and evaluate your core argument. Less is more [21]. Too much content also tempts presenters to speak faster, making the pitch difficult to follow. Prioritizing only the most essential and relevant elements ensures that your key messages remain clear and memorable [7].

Selected principles from storytelling can help improve the logical flow and accessibility of a funding pitch [4,9,22–29]. However, narrative elements should support rather than dominate the scientific argumentation and must not replace evidence, appropriate uncertainty, or transparent communication of limitations and risks.

## Rule 6 – Craft your visual presentation

The use of visual media in funding pitches depends on the specific requirements of the funding organization, the presentation setting, and the time available. Most funding pitches will involve a classical presentation with visual slides, commonly using PowerPoint or similar software. Where appropriate, other forms of visualization such as physical models, prototypes, or demonstration objects may also support your argument, particularly when they make a novel technology, method, or application easier to understand. Whatever format you choose, every visual element should have a clear purpose and support your message, not replace it [30]. When preparing and polishing your slides, follow established guidelines for effective scientific figure and image design and avoid typical pitfalls (Fig 6) [7,30–39]. Especially in short funding pitches, a graphical abstract or overview figure of the project can help the review panel quickly understand the central concept, research strategy, or relationship between individual work packages [36,40].

**Fig 6:**
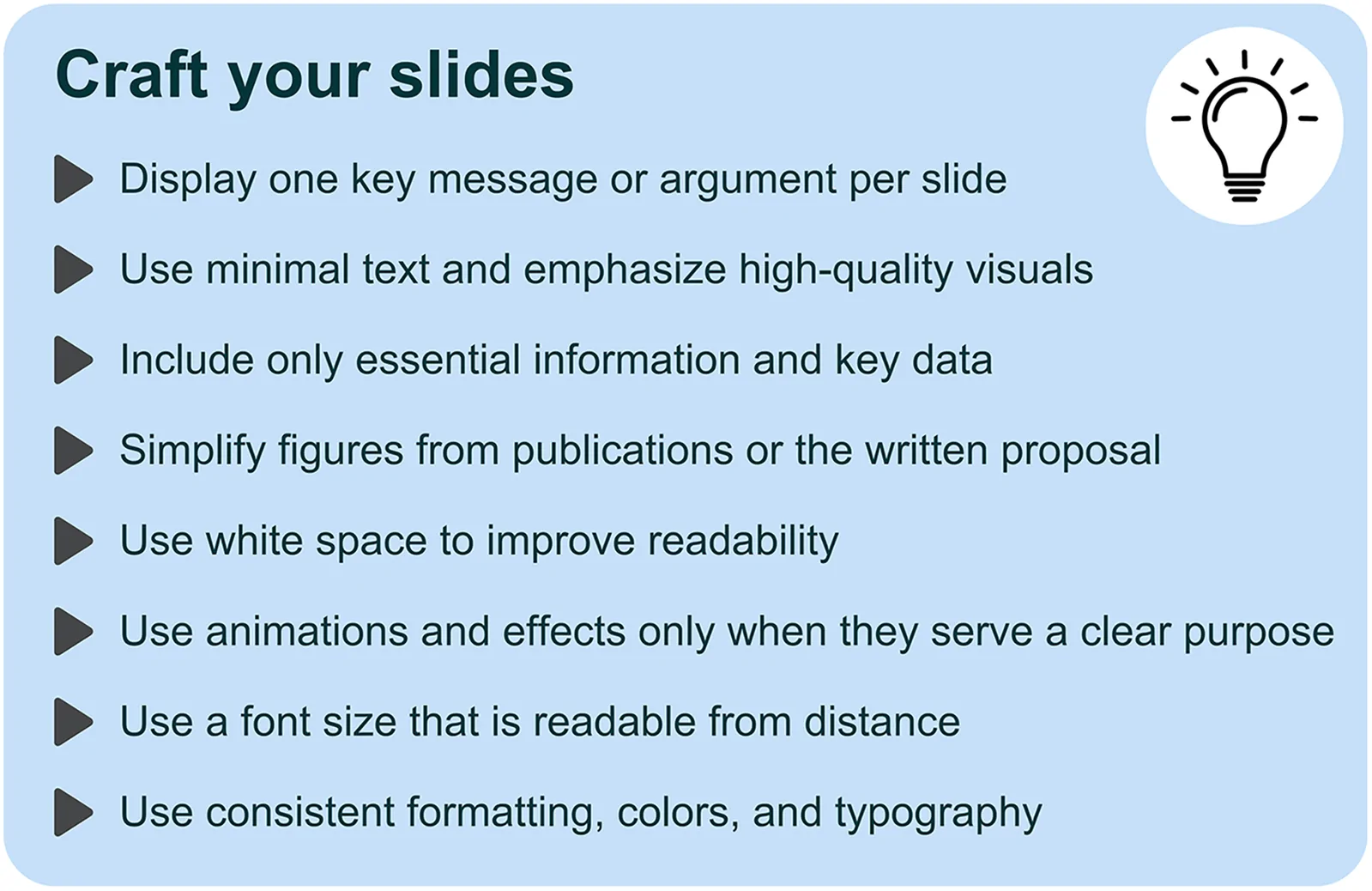
How to craft effective presentation slides.

Avoid simply copying figures from publications or the written grant proposal as these are often too complex to be processed within a few seconds. Take your time to simplify them, focus on the information that directly supports your key message, and avoid “chart junk” [30]. If you need to display complex info, such as your research strategy or experimental workflow, reveal it step by step in a logical order to keep the review panel oriented [38]. Finally, ensure a clear connection between your spoken and visual presentation: talk about what you show, and show what you talk about [41]. Visuals that are not directly connected to your argumentation can distract from the message rather than strengthen it. Test the slides on different screens and, if possible, check with a test audience to ensure that the essential information can be understood quickly [33,38].

## Rule 7 – Get ready for a productive discussion

Your funding pitch does not end with your last sentence. The subsequent discussion is an integral part of the evaluation process and requires equally careful preparation. Reviewers may use their questions to explore different aspects of your proposal, clarify uncertainties, and test how well you understand the strengths, limitations, and feasibility of your project. Regardless of the nature of their questions or objections, you must respond professionally and confidently (Table 3).

**Table 3:** Common questions and response strategies in funding pitch discussions.

Situation	Response
Scientific rationale or relevance is questioned	Refer to the scientific rationale, connect it to the problem or knowledge gap, and support your argumentation with relevant evidence.
Methodology or limitations are questioned	Acknowledge the limitation, explain why the chosen approach is appropriate, and provide supporting data or methodological justification where available.
Feasibility, timeline, or resources are questioned	Explain why the project is realistic within the proposed time and resources and refer to preliminary work, available expertise, infrastructure, or established collaborations.
The reviewer is not convinced by your preliminary data or evidence	Clarify what the data demonstrate, show additional data on backup slides if helpful, and/or refer to relevant published studies.
Budget or requested resources are questioned	Explain why the requested resources are necessary for the proposed work and relate them directly to specific project activities or objectives.
Risks or contingency planning are questioned	Acknowledge the risk and explain the alternative strategy or contingency plan you have prepared if the original approach does not work as expected.
You do not know the answer or the question is outside your expertise	Be transparent and avoid speculation. Apply the “offer & refer” principle: provide what you know and, where appropriate, refer to a colleague, relevant study, or additional information that can be provided later if permitted.
You do not fully understand the question	Rephrase the question before answering: “Did I understand you correctly that you would like to know more about …?”
You receive unexpected or strong criticism	Acknowledge the concern, clarify the underlying criticism, respond to the scientific issue directly, and explain how the concern is addressed in the project. Avoid becoming defensive.

Anticipate potential questions by carefully reviewing your proposal from the perspective of the review panel. Identify assumptions, limitations, risks, or aspects that may require further explanation, and make sure you are familiar with relevant literature, current developments, competing positions, and emerging concepts in your field. Collect supporting data, facts, and illustrative examples. Additional backup slides can provide further details when requested, but essential evidence and key arguments should already be part of the funding pitch itself.

However, even with the best preparation, you may encounter questions for which you don’t know the answer or that fall outside your expertise. In these situations, avoid speculation and communicate openly what you can and cannot answer. You may apply the “offer & refer” principle by providing the information you have and referring to a relevant study or, where appropriate, to a knowledgeable colleague in the room. If the review procedure permits follow-up information, you may also offer to provide additional details later [38]. If you do not fully understand a question, repeat and rephrase it to confirm understanding and avoid misinterpretation [20,37]. When facing critical comments, first acknowledge the reviewer’s concern and address it directly. If the criticism identifies a real limitation or risk, explain how it is considered in your project and, where appropriate, describe alternative approaches or contingency plans. Supporting data and additional arguments can help clarify your reasoning, but avoid becoming defensive or simply shifting attention away from the concern. Above all, communicate respectfully and professionally and treat critical questions as part of the scientific evaluation rather than as a personal attack. A clear and composed discussion can further demonstrate your understanding of the project and your ability to conduct it successfully.

## Rule 8 – Practice, rehearse, and get feedback

Both your funding pitch and the discussion should be practiced before the actual presentation. When rehearsing, your test audience should ideally include colleagues from both within and outside your specific research field [7,21,38]. If possible, conduct at least one run-through under almost realistic conditions and ask your audience for honest, constructive, and targeted feedback. Time your pitch and identify unnecessary content if you exceed the allowed time. Encourage your test reviewers to ask challenging questions and discuss appropriate answers [15,16]. If you tend to speak too quickly, practice the talk at a pace that feels excessively slow.

For larger research consortia or collaborative proposals, joint rehearsals are particularly important. Ideally, the complete pitch and discussion should be practiced at least once with all involved speakers, including transitions between presenters, timing, technical setup, and the handling of questions. Carefully selected colleagues can act as a mock review panel and challenge the consortium from different scientific perspectives.

While practicing, make sure that your key messages are fully understood. Make use of simple terms and analogies [2]. After your test pitch, ask the audience: *“In one sentence, what is your takeaway from my talk?”* If the response does not align with your intended message, revise your pitch. Any noise that interferes with clear communication should be identified and removed. This may include:

- Jargon overload or assumed knowledge,
- Dense slides or unreadable visuals,
- Overly complex graphs,
- Lack of focus or poor transitions,
- Monotone voice or low engagement.

Finally, practice alone several times before and after working with a test audience [42]. Speak aloud rather than rehearsing only in your head and practice standing up. Recording yourself on audio or video can help identify issues with body language, voice, pauses, speaking pace, and slide transitions [4,21]. If useful, speech-to-text or AI-based tools may additionally help analyze a transcript for filler words, repeated phrases, or unnecessarily complex formulations. These tools are optional and should complement, rather than replace, your own review and feedback from colleagues. The goal of rehearsal is not to memorize a perfect script, but to build clarity, confidence, and flexibility for both the pitch and the discussion.

## Rule 9 – Prepare yourself

Having accurate information about the setting and format of your funding pitch helps avoid unforeseen disruptions and allows you to feel more confident and to focus on the presentation itself. Carefully review all instructions provided by the funding organization and clarify uncertainties in advance. This includes the exact presentation and discussion time, required or restricted content, number or format of slides, submission deadlines, technical setup, whether additional backup slides or other presentation materials are permitted.

Familiarize yourself with the practical setting whenever possible [4]. Find out whether you’ll be speaking from a lectern, standing freely, or participating online, whether the discussion will be moderated, and whether slides will be controlled by you or the organizers. If your slides must be submitted in advance, check the final version carefully and keep an additional copy available in a compatible format. In online settings, test your camera, microphone, internet connection, and screen sharing in advance. Plan sufficient time before the session and, if possible, test the technical setup under realistic conditions. For collaborative funding pitches, make sure that all presenters know the sequence, transitions, and technical procedures.

Finally, prepare yourself mentally for the presentation and discussion. Nervousness is a natural response to an important evaluation situation [9,43]. Rehearsal, familiarity with the setting, light exercise, and simple relaxation or breathing techniques can help reduce unnecessary stress [4,9,38,44]. Focus on communicating your project clearly, responding thoughtfully to the review panel, and relying on the preparation you have already completed.

## Rule 10 – Deliver an effective pitch

Even the best structure and the strongest arguments can lose impact if they are delivered unclearly. When presenting your funding pitch, remember that your goal is not to perform, but to communicate clearly, confidently, and credibly to the review panel [37].

Trust your preparation. You have practiced, received feedback, and refined both your pitch and your responses to potential questions. Convey enthusiasm and authenticity, stay focused and maintain professionality during the discussion [3,42,45].

Use your *voice* strategically [37]. Variations in speed, volume, and pitch can emphasize key statements and help maintain attention [45]. Avoid speaking monotonously or too quickly and include short, intentional breaks in your talk to emphasize key points and to give the review panel time to process your arguments [45,46].

Make use of open and natural *body language* [45]. Stand stable, keep your hands visible, and use hand gestures to emphasize statements, illustrate contrasts, or enumerate arguments [8]. Moderate moving can add dynamics but avoid restless pacing. Maintain eye contact with the review panel rather than speaking to the screen or reading from your slides [7,37,44,47].

Humor may occasionally help create connection or illustrate a point, but it should be used carefully and only when appropriate for the audience, topic, and setting [48].

Above all, create coherence between your scientific argumentation, your visual presentation, your voice, and your body language [18,46].

## Conclusion

Funding pitches provide applicants with an opportunity to present and discuss their proposed research as part of a competitive funding decision. Effective preparation requires more than simply condensing the written proposal into a short presentation. Applicants need to identify the aspects most relevant to the review panel, define clear key messages, explain the scientific problem and proposed approach, select strong evidence-based arguments, and organize these elements within a clear and focused structure. Visuals, voice, and body language should support the scientific message and help reviewers follow the argumentation. Moreover, the discussion is an integral part of the funding process and requires careful preparation to address questions, concerns, and critical comments professionally and confidently.

Finally, practice and constructive feedback are essential for refining both content and delivery. The ten rules presented here provide a practical and adaptable framework for preparing, rehearsing, and delivering funding pitches and for discussing the proposed project confidently and professionally with a review panel (Fig 7).

**Fig 7:**
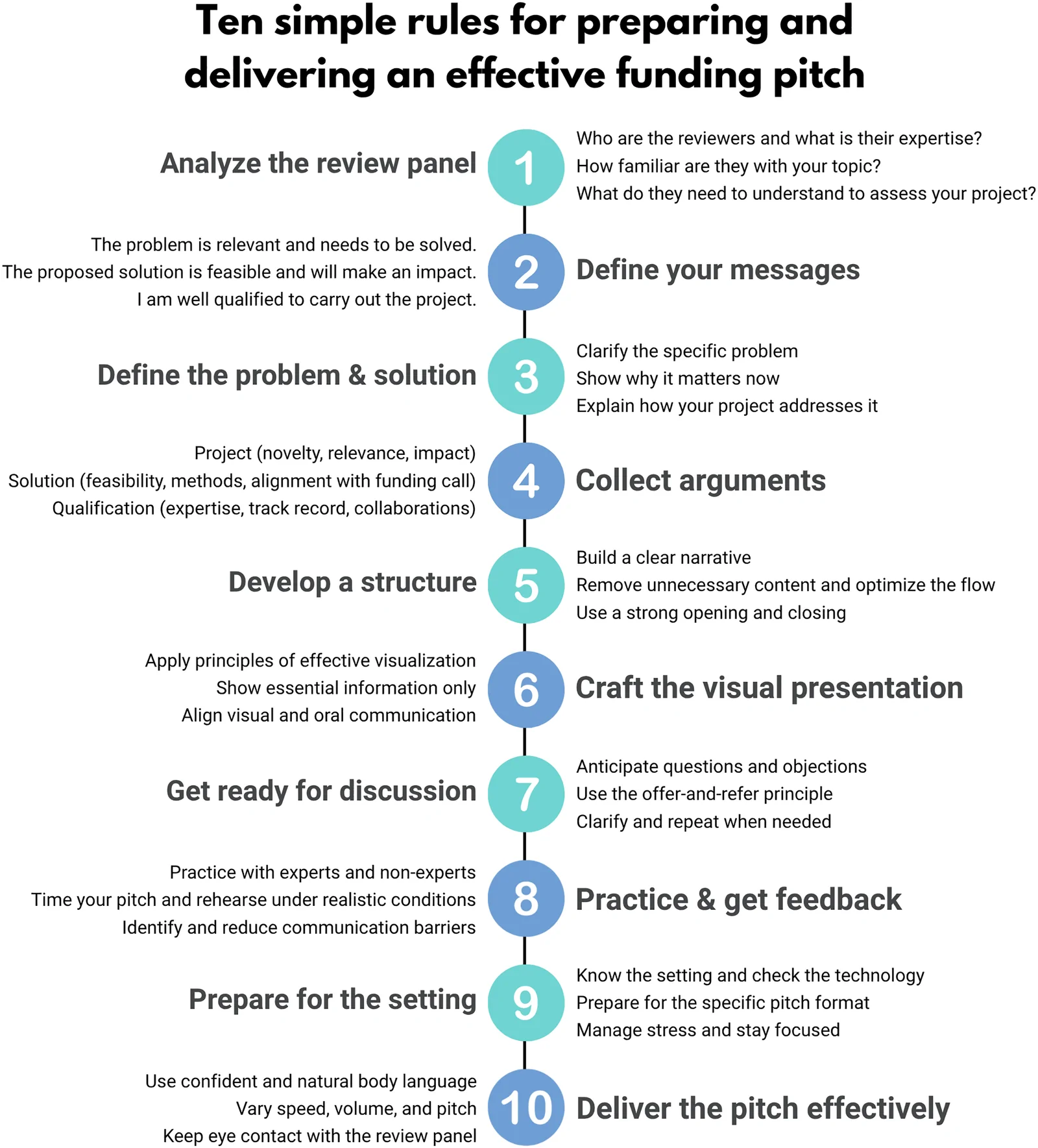
Ten simple rules for effective funding pitches.
